# The Influence of the Tongue on the Development of Dental Malocclusion

**DOI:** 10.7759/cureus.61281

**Published:** 2024-05-29

**Authors:** Mrunali Deshkar, Nilima R Thosar, Sakshi P Kabra, Ramakrishna Yeluri, Nilesh V Rathi

**Affiliations:** 1 Pediatric and Preventive Dentistry, Sharad Pawar Dental College and Hospital, Datta Meghe Institute of Higher Education and Research, Wardha, IND; 2 Pediatric Dentistry, Sharad Pawar Dental College and Hospital, Datta Meghe Institute of Higher Education and Research, Wardha, IND; 3 Pediatric Dentistry, Dr. D. Y. Patil Dental College and Hospital, Dr. D. Y. Patil Vidyapeeth, Pune, Pune, IND

**Keywords:** macroglossia, myofunctional therapy, habit, malocclusion, tongue

## Abstract

The tongue supports the upper dental arch and encourages healthy dental arch development when it rests against the roof of the mouth. On the other hand, over time, malocclusion can result from incorrect tongue position, such as lying low in the mouth or thrusting forward during swallowing or speaking. As a muscular organ, the tongue applies forces to the jaws and teeth that may help with malocclusion or hinder it from aligning properly. The dentition and jaws grow and align according to the way the tongue, teeth, and surrounding structures interact. The tongue's morphogenetic function includes forming the arches and having an important impact on the maxillary complex's development. The tongue frequently assumes a balancing and compensatory function in subsequent phases, functioning more or less like a natural orthodontic bite. In adults, the tongue is able to compensate for problems like open bites, teeth that are out of alignment, or differences in the occlusal and sagittal planes of the spine. In this context, the tongue's ability to sustain occlusion during malocclusion can be considered a compensatory response. This is comparable to how lingual dysfunction may contribute to malocclusion or act as a potential source of recurring orthodontic instability. In order to diagnose and treat orthodontic issues, dental professionals must know the connection between tongue position and dental malocclusion. Malocclusion can be prevented or minimized with early intervention, such as myofunctional therapy to correct tongue position and habits, improving dental health and well-being overall.

## Introduction and background

The tongue is a primarily muscular organ that fills the functional area of the mouth. Composed of striated muscle tissue, it plays an active role in processes like sucking, chewing, swallowing, and speech production, all essential for maintaining a high quality of life [1]. The temporal and mandibular bones combine to form the temporomandibular joint (TMJ), which is also in charge of speech, swallowing, and chewing. Due to their same neuromuscular system, the scapular and cervical areas are primarily affected by alterations to it [2]. There is growing consensus among dentists towards the delicate and complex occlusion establishment and more emphasis is being laid on it. It involves not only how upper and lower teeth relate to one another, but also how teeth, muscles, and mandibular movement work together harmoniously. When the head and body posture are in harmony and are optimally matching, this is known as ideal occlusion [3,4].

The stomatognathic system, a crucial part of the upper body, plays a key role in postural control [5]. The immediate alteration of the functional body posture is one of the most important problems associated with malocclusion. Function, aesthetics, and biomechanics may all be affected [6,7]. Among several factors that can alter one's balance and posture, dental malocclusion is highly significant [8]. As the cranial position in the cervical area is recognized to be intimately correlated with the body's balance, this position is reflected in the body's overall balance [9]. The anterior cervical spine and TMJs hold the skull's centre of gravity. The head, neck, and shoulder girdle muscles are in charge of preserving the joint's upright posture. Therefore, postural imbalance may result from any alteration to one of these structures [10].

## Review

The tongue's anatomical relations

**Figure 1:**
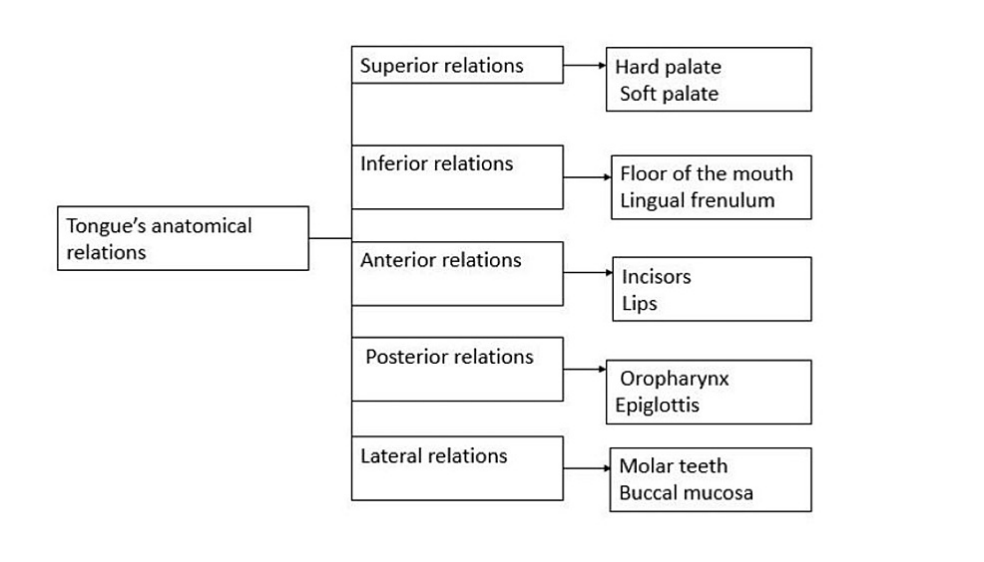
Tongue's anatomical relations Image created by the authors.

The somite mesoderm is the origin of the anatomic connections between the tongue muscle cells [11]. The muscle cells that migrate into the tongue from the occipital somites are the source of the lingual musculature. The tongue, the occipital region, and the hyoid bone, which originate from the second branchial arch, are closely interconnected both in terms of embryology and function. The tongue is anatomically connected by a number of pathways to the hyoid bone and the suprahyoid (SHM) and infrahyoid muscles (IHM) of the hyoid musculature. The tongue is attached to the hyoid muscles by the lingual septum and the hyoglossus membrane (Figure 1) [12].

Electromyography revealed electrical activity in the anterior belly of the digastric muscle and the omohyoid muscle during various tongue movements [10,13]. These muscles act as bridges to facilitate appropriate tongue-head (neck) association during cervical tract rotation, flexion, and extension [12]. The SHM and IHM work together to synchronize the movements of the jaw and tongue during the early stages of swallowing and speech production. For all tongue motions (apart from retraction), the suprahyoid and lingual musculature work in conjunction with the tongue. The pharyngeal gap widens when the hyoid bone moves craniocaudally during respiration, which is caused by the extrinsic tongue muscles [12]. The tongue's anterior region is generally thought to be important for non-respiratory functions and its posterior region is thought to be important for breathing [14]. The fact that both intrinsic and extrinsic muscle groups in the tongue always function in concert rather than in isolation should be underlined. Maintaining a balance in muscle tone is crucial to prevent dysfunctions that may alter the functioning of the tongue and the positioning of the hyoid bone. The tongue anatomically envelops the occipito-cervical zone and the front portion of the cervical neck, masticatory muscles, including the TMJ, and the three layers (superficial, middle, and deep) of the cervical fascia [15,14]. The tongue and the region above the hyoid bone are linked to muscles such as the jaw muscle masseter, cheek muscle (buccinator), temporalis, wing-shaped muscle (pterygoid), and lower jaw muscle (mylohyoid). The quadrilateral mylohyoid muscle structure forms the base of the oral cavity, which consists of two bellies. The anterior fascia of the digastric muscle is closely related to the lower fascial layers, which are situated above the geniohyoid and hyoglossus muscles [15].

The mylohyoid muscle connects to the hyoid bone through a sagittal fibrous lamina and a connecting raphe. The infrahyoid region, situated beneath the hyoid bone, extends upward into the suprahyoid area. The deep cervical fascia runs from the hyoid bone to the upper mediastinum, traversing the visceral area, the anterior cervical region, the posterior cervical region, the carotid region, the retropharyngeal region, and the perivertebral region [12,13]. Tongue muscles, the sternocleidomastoid muscle, and strap muscles are among the muscles attached to the thoracic outlet that provide fascial continuity in this region. The tongue's ability to resemble a hydrostat in both structure and function is a result of the complex synchronization between the central and peripheral nerve systems. To allow the tongue to function as intended, every muscle that comes into contact with the tongue, whether directly or indirectly, responds with an adequate contractile tone [13].

Functions of tongue

Mastication

The tongue's role in aiding with chewing is widely acknowledged. In addition to adjusting the food's position between the teeth with the help of the buccinators, it also turns and combines the food with saliva. However, it is clear that the tongue has a big impact on masticatory efficiency [16].

Swallowing

Environmental factors, the position and thrust of the tongue, mouth breathing, and non-nutritive oral habits have all been related to patterns of swallowing. Ankyloglossia, sometimes known as tongue tie, is a congenital oral abnormality that causes changes in swallowing and disrupts the stomatognathic system's growth and development. Sometimes, children who swallow improperly fail to touch the front of the palate with the tip of their tongue. Furthermore, there is a connection between the position and mobility of the mandible and the lingual muscle action [17]. A physiological swallowing process depends on the function and posture of the tongue. Atypical or incorrect lingual postures are consistently linked to facial deformities in children with deciduous and mixed dentition [18].

Speech

A speech disorder's complex multifactorial origins include genetics, which is the most well-established cause of speech disorders or abnormal articulation of one or more sounds. The development of spoken language involves a complex coordination of the neural pathways controlling the tongue, palate, teeth, lips, alveolus, and other vocal organs [19]. Thus, the ability of the body to produce sound waves that travel through the air and can cause the eardrum to vibrate is necessary for spoken language. The speech apparatus then reproduces these sounds by controlling the air stream, which includes the lung, larynx, and upper tract, which includes the throat, mouth, palate, and nose [19].

Breathing

Nasal breathing happens when the tongue is at rest. However, forced mouth breathing is always associated with a habit or a force. While physiological breathing is an active component of harmonious craniofacial development, its influence on the development of the skull can result in functional and skeletal alterations when external factors alter its mechanism [20]. Three types exist for breathing: nasal, mixed (oro-nasal), and oral. Alterations in the skeletal and functional development of the orofacial region are associated with oral breathing, a parafunctional habit in which air passes entirely or partially through the mouth rather than the nose. Thus, it makes perfect sense that a changed breathing pattern, for example, the habit of breathing through the mouth instead of the nose can impact the positioning of the head, jaw, and tongue [20]. Maintaining these postural changes would have three expected effects on growth: an increase in anterior face height and super-erupting posterior teeth; the mandible rotating down and back, which opens the bite anteriorly and increases overjet; and vertical growth abnormalities of the ramus, combined with increased pressure from stretched cheeks, leading to a narrower maxillary dental arch [20].

Tongue size and malocclusion

**Figure 2:**
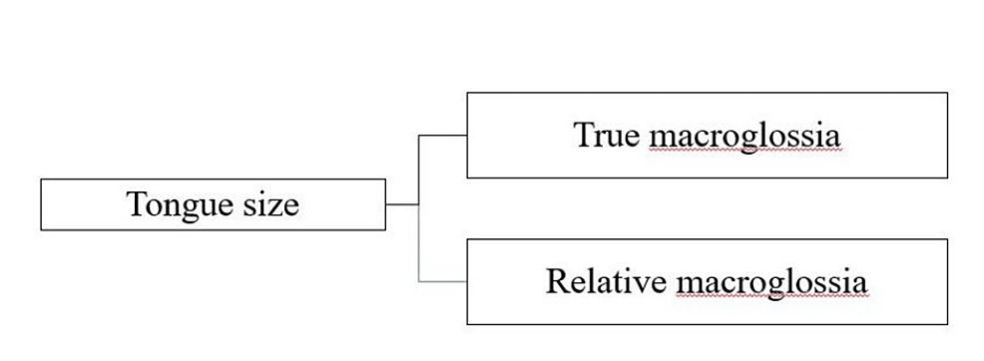
Types of macroglossia Image created by the authors.

According to Boucher, macroglossia is defined as an enlarged tongue caused by endocrine problems, tumors, or muscular hypertrophy [21]. A tongue's normal size varies with age, growing at its fastest rate during the first eight years after birth and reaching its maximum size at 18. According to Martínez et al., macroglossia is a long-term, painless enlargement of the tongue that extends past the alveolar ridge or teeth [22]. The classification of tongue size and oral cavity dimensions can be categorized into two groups: true macroglossia and relative macroglossia, depending on various factors such as tongue size and other oral cavity components (Figure 2) [23,24].

True Macroglossia

True macroglossia is characterized by the actual increase of the tongue as a result of an underlying illness. This can be caused by various factors such as inflammation, tumors, vascular malformations, metabolic disorders, or congenital abnormalities affecting tongue development. The underlying cause can frequently be determined by observing pertinent histological results [24].

Relative Macroglossia

This condition is called relative macroglossia when the tongue seems larger than normal in comparison to other aspects of the oral cavity. Smallmouth cavities, underdeveloped lower jaws known as micrognathia, and disorders affecting mouth muscles' tone and coordination can all contribute to this [25].

Conditions Related to Macroglossia

Down syndrome: Children with Down syndrome frequently have relative macroglossia, a condition in which there are abnormalities in craniofacial development and hypotonia (low muscular tone), which causes the tongue to appear bigger [25].

Pierre Robin syndrome: This syndrome is identified by the coexistence of micrognathia (a small lower jaw) and glossoptosis (the downward displacement or retraction of the tongue) and cleft palate. The tongue may appear relatively large due to the smaller size of the oral cavity and jaw [25].

Diagnosis

The diagnosis is often established through assessment of subjective criteria like tongue shape and protrusion, speech impediments, swallowing issues, or respiratory challenges. The protrusion of the tongue through the lips is the primary indicator of macroglossia. When the enlarged tongue is palpated, it appears clinically normal, and tongue pressure has caused the alveolar bone to thin [26].

Effect on Malocclusion

Protrusion of the tongue may result in diastemas, forward inclination of the upper and lower incisors, and anterior open bite. Additional clinical findings in patients with macroglossia may include TMJ disorder and maxillofacial issues. Macroglossia can result in various clinical complications, including noisy breathing, respiratory problems like upper airway obstruction, challenges with feeding that result in malnutrition, as well as infections affecting the tongue due to prolonged exposure to air. Protruding the tongue can contribute to the development of skeletal class III disharmony, an enlarged gonial angle, and an anterior open bite, all of which can impact skeletal growth [26].

Tongue thrust and malocclusion

Tulley defined tongue thrust as the position of the tongue between the dental arches that happens when the tongue tip slides forward between the teeth to touch the lower lip during speech or during tooth development. This condition leads to an open bite and the forward positioning of the front teeth segment [27].

Etiology

Genetic or heredity factor: Tongue thrust can be caused by specific anatomical or neuromuscular variations in the orofacial region. For example, the hypertonic orbicularis oris activity [28].

Learned behaviour: One can develop the habit of tongue thrusting. A few risk factors that can result in tongue-thrusting include poor bottle feeding technique, extended thumb suckling, recurrent tonsil infections and upper respiratory tract infections, and a prolonged period of gum or tooth discomfort may cause a person to adjust their swallowing technique to relieve pressure on the affected area [28].

Infections: The tongue is forced forward due to discomfort and a reduction in space, resulting in a tongue thrust swallow, which causes chronic tonsillitis, allergies, and upper respiratory tract infections, including mouth breathing. The physiological necessity of maintaining a sufficient airway may also be the reason for its existence [29].

Feeding practices: A contributing cause of tongue thrusting has been identified as prolonged bottle feeding and an improper swallowing pattern.

Types of tongue thrust

Physiologically, the standard tongue-thrust swallow observed in infants may persist as a habitual behavior even after corrective measures are taken for a malocclusion. When this tongue-thrusting behavior becomes functional, it serves as an adaptive response aimed at creating an effective oral seal. Anatomic factors, such as having a larger-than-average tongue, can contribute to individuals adopting an anterior tongue posture, further reinforcing the persistence of the tongue-thrust swallow pattern [30].

Classification of Tongue Thrust

**Figure 3:**
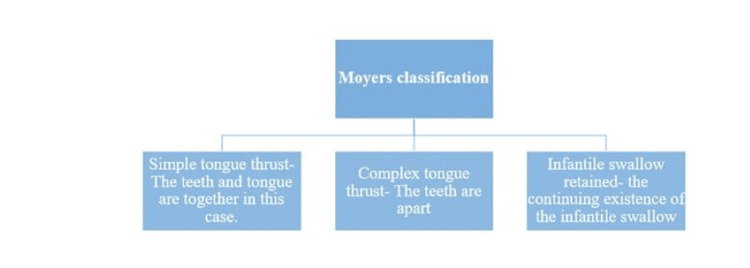
Moyers' classification

Moyers' classification is illustrated in Figure 3 [26].

Intra-Oral Features

Increased overjet is due to proclined, spaced, and occasionally flared upper anterior teeth. The positioning of the lower front teeth can vary, either being retroclined or proclined, and this variation is determined by the type of tongue thrust involved [31]. In order to establish an anterior lip seal, the tongue protrudes forward during the process of swallowing [31]. An anterior open bite is observed [32]. Atypical teeth contact during the swallowing movement is a characteristic of the basic tongue thrust. Unlike complex tongue thrusts, they show good intercuspation of the posterior teeth.

Extra-Oral Features

Some of the extra-oral features are increased vertical dimension of the lower anterior face; a face exhibiting dolichocephaly, characterized by an elongated cranial vault and facial structure; at rest, lips are unable to completely close together or seal. The face appears devoid of expression during swallowing because it's the facial muscles, not the masticatory muscles that stabilize the mandible. There are difficulties with speech, such as lisping and sibilant distortions. Muscle activation in the mentalis appears abnormal [30].

Diagnosis

Assessing for upper respiratory tract infections, digit-sucking habits, and neuromuscular issues, and examining the swallowing patterns in siblings and parents are part of the process to rule out potential factors, including hereditary ones [33,34].

Management

**Figure 4:**
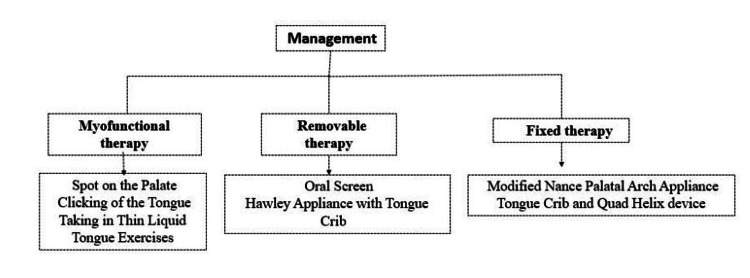
Management protocol

According to the 2005 report from the American Academy of Pediatric Dentistry Council on Clinical Affairs, addressing tongue thrust may require a combination of approaches including myofunctional therapy, habit control strategies, habit-breaking devices, orthodontic treatments, and potentially surgical procedures (Figure 4) [35].

Myofunctional therapy

The goal of orofacial myofunctional therapy is to improve the proprioceptive capacities, muscle tone, and general mobility of the muscles in the neck and face through specific exercises [36]. Orofacial myofunctional therapy is centred around the re-education and improvement of neuromuscular function in the muscles involved in swallowing, tongue control, nasal breathing, and the natural resting positions of the lips, tongue, and cheeks [37].

Spot on the Palate

Children are asked to identify a specific location on the palate, which is usually behind the upper incisors. After that, they are instructed to repeat this motion 10 times, making sure the tongue stays in the same spot for 10 seconds each time. The purpose of this exercise is to improve proprioception and oral motor control, which will help with speech and swallowing development [38].

Clicking on the Tongue

A characteristic popping or clicking sound that is frequently audible close to the front of the mouth can be produced by pressing the tongue up against the roof of the mouth. The sound of the click is produced by the release of air that has been trapped between the palate and the tongue. In certain languages, it's a standard method or perhaps something people do on a daily basis.

Taking in Thin Liquid

Children can improve their oral muscle control and coordination by keeping water in their mouth and stabilizing their tongue briefly before swallowing. This practice encourages good swallowing mechanics, and it might be suggested as a component of programs for developing oral motor skills or speech therapy.

Tongue Exercise to Promote Lateral Tongue Movement

The tongue exercise for promoting lateral tongue movement involves drawing out the tongue and moving it from extreme left to right, holding each position for 10 seconds. This movement is repeated 10 times on both sides to strengthen and improve the flexibility of the tongue muscles, aiding in various oral functions such as speech and swallowing [39].

Exercise for Rolling the Tongue

The child has to roll his or her tongue, folding its edges in toward the middle to make it look like a taco shell. The child must roll over and extend their tongue as far as they can, holding it for 10 seconds at a time, then repeating the process 10 times.

Push the Tongue and Grasp the Tongue Blades

A child is tasked with holding two tongue blades or ice cream sticks using their lower front teeth, ensuring that two to three centimeters of the sticks are visible outside their mouth. The child then tries to lift the sticks, encountering resistance from the firmly held blades [39].

Tongue Retraction

The tongue retraction exercise involves the patient pressing the palate firmly with the back of the tongue and holding this position for a minimum of three seconds. It's recommended to repeat this exercise five times during a session to strengthen the tongue's ability to retract properly, aiding in various oral functions such as proper swallowing and speech articulation [38].

Removable appliance therapy

The Oral Screen is a myofunctional appliance that creates a barrier between the surrounding muscles and the teeth. It involves applying a fine layer of processed acrylic material over occluded casts, extending deeply into the vestibular sulcus on both the labial and buccal surfaces. On the other hand, the Hawley Appliance with Tongue Crib utilizes components made from 0.7 mm Hard Stainless Steel Wire (HSSW), including the labial bow, a crib, and the Adam's clasp [7]. The cast is initially covered with a separating medium, allowed to dry, and then used to create an acrylic plate using the sprinkle-on technique [40].

Fixed appliance therapy

The modified Nance Palatal Arch appliance incorporates an acrylic button to assist in positioning the tongue correctly [41]. The other appliance design includes a combination of a Tongue Crib and Quad Helix device, featuring a stainless steel wire with a thickness of 0.036 inches soldered onto bands fixed to the first permanent molars. This integrated appliance is specifically utilized to address and rectify tongue-thrusting behavior, as well as to tackle associated issues related to transverse, vertical, and functional problems within the oral cavity [42].

## Conclusions

The position of the tongue is very important for maintaining good dental alignment and occlusal harmony from early childhood into adulthood. Over time, maladaptive tongue postures like tongue thrusting or macroglossia can cause malocclusion by altering the delicate balance of forces inside the oral cavity. For harmonic dental occlusion, the tongue position must be maintained in a balanced manner while at rest and when swallowing. To get the best possible treatment results for patients with malocclusion, clinicians should think about evaluating and treating tongue position. Furthermore, dealing with complex cases of malocclusion associated with tongue-related disorders requires coordinated efforts between orthodontists, speech therapists, and other healthcare specialists.
